# Who Benefits from Chronic Opioid Therapy? Rethinking the Question of Opioid Misuse Risk

**DOI:** 10.3390/healthcare4020029

**Published:** 2016-05-25

**Authors:** Elizabeth Huber, Richard C. Robinson, Carl E. Noe, Olivia Van Ness

**Affiliations:** The University of Texas Southwestern Medical Center at Dallas, 5323 Harry Hines Blvd., Dallas, TX 75390, USA; elizabeth.huber@utsouthwestern.edu (E.H.); carl.noe@utsouthwestern.edu (C.E.N.); Olivia.vanness@utsouthwestern.edu (O.V.N.)

**Keywords:** chronic pain, chronic low back pain, opioids, chronic opioid therapy, biopsychosocial approach

## Abstract

Beginning in the late 1990s, a movement began within the pain management field focused upon the underutilization of opioids, thought to be a potentially safe and effective class of pain medication. Concern for addiction and misuse were present at the start of this shift within pain medicine, and an emphasis was placed on developing reliable and valid methods and measures of identifying those at risk for opioid misuse. Since that time, the evidence for the safety and effectiveness of chronic opioid therapy (COT) has not been established. Rather, the harmful, dose-dependent deleterious effects have become clearer, including addiction, increased risk of injuries, respiratory depression, opioid induced hyperalgesia, and death. Still, many individuals on low doses of opioids for long periods of time appear to have good pain control and retain social and occupational functioning. Therefore, we propose that the question, “Who is at risk of opioid misuse?” should evolve to, “Who may benefit from COT?” in light of the current evidence.

## 1. Introduction

Beginning at the turn of the millennium, questions arose about the under-utilization of opioid pain medication to treat individuals suffering from chronic noncancer pain (CNCP) [1]. Terms such as “opiophobia” became used more widely and referenced a potentially irrational fear that providers had with regard to utilizing this class of medication [2]. With the support of pharmaceutical companies, many pain management physicians invested time and energy to educate the public and their peers that opioids could be used safely and effectively for CNCP, including chronic low back pain (CLBP) [1]. One focus that developed out of this movement was to identify those who were at risk for opioid misuse as well as to develop interventions for those who may have succumbed to addiction [3]. Measures such as the Pain Medication Questionnaire (PMQ) [4] and The Screener and Opioid Assessment for Patients with Pain (SOAPP) [5] were developed, as well as guidelines for urine drug testing, to identify those who were at risk of misusing their opioids or to determine who was engaging in aberrant, and potentially harmful, opioid use.

Since that time, the evidence for the efficacy of chronic opioid therapy has not grown in a substantial, or even discernible, manner [6,7]. Rather, evidence accumulated regarding the potential harmful effects of opioids, including substance use disorders, endocrinopathy, opioid-induced hyperalgesia, and death [7]. In fact, in a recent review of the use of chronic opioid therapy (COT) for chronic pain by Chou, Turner *et al.* [7] concluded, “Evidence is insufficient to determine the effectiveness of long-term opioid therapy for improving chronic pain and function. Evidence supports a dose-dependent risk for serious harms.” (p. 276).

Despite these findings, opioids continue to be used for CNCP [8]. However, limited research has been conducted on those individuals who remain on low doses of opioids (e.g., 10–20 mg morphine equivalent) with adequate pain control and functioning. Therefore, we propose that the question should evolve from, “Who is at risk for opioid misuse?” to, “Who may benefit from COT?” given the current evidence for this intervention. 

## 2. Chronic Pain

### 2.1. The Scope of the Problem

The International Association for the Study of Pain (IASP) [9] defines pain as “an unpleasant sensory and emotional experience associated with actual or potential tissue damage” and chronic pain as “pain persisting beyond the normal expected time of healing,” which is typically considered pain that lasts for three to six months or more [10]. According to the Institute of Medicine (IOM) [11], approximately 100 million adults are afflicted by chronic pain in the United States. Gatchel, Peng, and colleagues [12] estimated that chronic pain accounts for more than 80% of physician visits. Furthermore, chronic low back pain (CLBP) is the most common chronic pain condition [11]. 

Pain is a costly condition, not only in terms of healthcare expenditures to treat chronic pain patients, but in terms of lost productivity and compensation for disability as well [13]. Furthermore, chronic pain is frequently associated with significant comorbid psychiatric conditions and emotional suffering [12]. It is difficult to measure the economic impact of chronic pain in the United States, as most studies focus on individual pain disorders rather than providing composite estimates across a broader range of common pain conditions [14]. Nonetheless, Gaskin and Richard [15] used the Medical Expenditure Panel Survey (MEPS) [16] to estimate the portion of U.S. health care costs attributable to pain, as well as the incremental annual costs associated with lower worker productivity as a result of pain. Their findings indicate that the annual costs of treating pain in the United States were greater than the costs of treating heart disease, cancer, and diabetes combined [15]. Gaskin and Richard [15] found that the total costs of treating pain annually fell somewhere between $560 and $635 billion in 2010 dollars, with additional health care costs due to pain ranging from $261 to $300 billion. They estimate the value of lost worker productivity due to pain ranged from $299 to $335 billion [15]. 

### 2.2. The Biopsychosocial Model

Given the high costs associated with treating chronic pain, it is not surprising that a significant amount of research has been conducted to better understand the causes and thus develop more effective methods to treat patients with pain. The biopsychosocial model is a theoretical approach that attempts to address health and illness based on a combination of biological, psychological, and social variables [17]. Underlying this model is the principal that all health-related issues, including pain, arise from a multifaceted interaction among these three factors. Research has demonstrated that it is not always possible to find a purely physiological cause in many cases of chronic pain; in particular, providers often struggle when reports of pain do not appear commensurate with identifiable physical pathology [18]. Prior to the development of the biopsychosocial model, physicians and researchers relied predominantly on a biomedical approach, which ascribes the etiology of disease, including pain, to biologic factors [17]. It was widely accepted among practitioners that the scope of the traditional biomedical model was too narrow to adequately address the complex processes that contribute to the condition of chronic pain [17]. A history of psycholosocial research has demonstrated the importance of emotional, behavioral, and cognitive factors that contribute to the perpetuation, and possibly the development, of chronic pain [19]. The authors propose that the biopsychosocial model represents the best current approach, one that is broader and better suited to address the multifaceted nature of pain, toward understanding and addressing chronic pain as an illness. The development of interdisciplinary pain management programs is largely attributable to the adoption by physicians of the biopsychosocial model to treat pain. 

The evidence for the effectiveness of interdisciplinary pain management programs is substantial. For instance, Mayer, Gatchel *et al.* [20] conducted a two-year prospective study comparing participants of an interdisciplinary functional restoration group to a standard treatment group. At two years, 87% of participants in the treatment group had returned to work compared to 41% of the control group. Furthermore, individuals in the treatment group had twice as many surgeries and significantly more health care visits. Similar results on the effectiveness of interdisciplinary treatment have been found in the U.S. and abroad [21,22,23,24,25]. In an investigation of the efficacy of multidisciplinary pain centers (MPCs), Turk and Okifuji [26] concluded that, not only does the MPC approach yield better results for patients in terms of the reduction in pain, emotional distress, and the use of analgesic medication as compared to alternative medical and surgical treatment options, MPCs can also save billions of dollars in health care expenditures. From an intervention perspective, the biopsychosocial approach emphasizes the importance of treating the initiating and maintaining biological and psychosocial factors of chronic pain. From an assessment perspective, the biopsychosocial approach emphasizes the understanding of the biological, emotional, social, and cultural contributors to pain and defines success, broadly, as adequate functioning in these areas. Therefore, the authors propose that the biopsychosocial model represents the best theoretical framework for determining who benefits from COT. 

## 3. Opioids to Treat Chronic Pain

According to the Centers for Disease Control and Prevention (CDC) [27], “In 2012, health care providers wrote 259 million prescriptions for opioid pain medication, enough for every adult in the United States to have a bottle of pills” (p. 1). Currently, evidence exists for pain relief from opioid therapy for short periods of time (approximately 16 weeks) [27]. For instance, Furlan, Sandoval *et al.* [28] evaluated 41 randomized control trials that investigated the effectiveness of opioids for a variety of pain conditions over a 5- to 16-week period and found that opioids outperformed placebos with regard to pain and functioning for individuals with neuropathic pain and fibromyalgia. Kalso, Edwards *et al.* [29] found similar results for the short-term use of opioids for musculoskeletal pain and neuropathic pain. More recently, Sander-Kiesling [30] followed 379 participants for one year in an open-label study after the conclusion of a randomized control trial and reported continued effectiveness of the combination of prolonged release oxycodone and naloxone. 

With regard to the development of addiction, Noble, Treadwell *et al.* [31] reviewed 26 studies, which consisted of a single randomized controlled trial and 25 long-term, uncontrolled trials. The authors noted that only 0.27% of the participants demonstrated evidence of addiction. Although the evidence was rated as “weak,” individuals who tolerated opioids showed clinically significant pain relief [31]. In a comprehensive review of the literature, only 3.27% of individuals with chronic pain were reported as developing abuse or addiction after exposures to COT, and approximately 12% of the studies reviewed found aberrant drug related behavior with regard to prescribed opioids [32]. However, limited research has been conducted to examine which individuals are likely to benefit from COT with more attention being given to who is at risk for addiction or pain medication misuse. 

Opioid prescription use has grown more controversial among physicians, elected officials, and the public in light of potentially harmful effects, primarily including physical dependence and psychological addiction [1]. Furthermore, there has been a rise in recent years in opioid abuse, between 1999 and 2012, the CDC estimates there was a 300% increase in overdose deaths related to opioids [8]. The CDC estimates that for every death in 2010 resulting from opioid overdose, there were: 733 nonmedical users of opioids, 108 people with abuse/dependence on opioids, 26 emergency room visits related to opioid misuse or abuse and 10 opioid abuse treatment admissions [8]. 

In addition to the risk of abuse, other common side effects of opioid use include sedation, dizziness, nausea, vomiting, constipation, physical dependence, tolerance, and respiratory depression [33]. Factors that have been shown to put patients at a higher risk for opioid abuse include being under 65 years of age, having a previous history of opioid abuse, people taking high daily doses of opioids, low socioeconomic status and those living in rural areas, Medicaid populations, patients with a history of depression, anxiety, posttraumatic stress disorder or childhood sexual abuse, and those taking psychotropic medications, other central nervous system depressants, or illicit drugs [8,34]. 

Beyond addiction, COT has been associated with unintentional overdose, fractures, myocardial infraction, endocrinological changes, and motor vehicle accidents [7]. For instance, a cohort study of 9940 individuals receiving COT for CNCP identified 51 opioid overdoses and six deaths [35]. Risk of overdose increased by prescribed dose, with individuals on greater than 100 mg morphine equivalent having a 1.8% annual overdose rate. Increased risk of fractures was found to be greater for individuals prescribed opioids [36], and individuals prescribed 20 mg morphine equivalent or greater were at higher risk of motor vehicle accidents [37]. Despite these findings, the question remains as to who may benefit from COT. 

### 3.1. Does Anyone Benefit from Chronic Opioid Therapy? 

Although research citing the risks associated with COT is abundant, there is also a consensus that opioids may be a beneficial aspect of treatment for some individuals with chronic pain [6]. Studies have indicated that patients with specific types of pain, including osteoarthritis pain, diabetic neuropathy pain, and low back pain, may benefit from controlled COT [38]. Several factors have been identified as contributing to the success of long-term opioid use for this select group of chronic pain patients, including appropriate informed consent and development of an individualized opioid management plan, proper initiation and titration of medication, careful monitoring throughout treatment including dose escalations, and management of breakthrough pain [6,39]. Indeed, the American Pain Society has issued clinical guidelines to assist physicians in prescribing chronic opioid therapy for CNCP [6]. Although the guidelines are useful once a physician has decided upon opioid use as an appropriate treatment method, limited evidence exists to pre-identify which particular chronic pain patients may benefit from COT.

### 3.2. The Case for Inclusive Screening Measures 

As a result of the increasing prevalence of opioid abuse and related complications and deaths, a number of measures have been developed to assist providers in identifying those chronic pain patients at a higher risk for opioid abuse or misuse. Such measures include the Screening Instrument for Substance Abuse Potential [40], the Prescription Abuse Checklist [41], the Prescription Drug Use Questionnaire [42], the Pain Assessment and Documentation Tool [43], and the Pain Medication Questionnaire [4]. The Screener and Opioid Assessment for Patients with Pain (SOAPP) and its revised version, the SOAPP-R, were developed to assess suitability of COT for patients with chronic pain based on the similar goal of identifying and excluding those patients most at risk for substance abuse [5]. Although all of these instruments may be effective to varying degrees at identifying which patients have a higher likelihood of abusing opioids, they all may be viewed as exclusion measures such that they are meant to identify those patients who should be excluded from opioid treatment. One of the inherent limitations of these measures is that, while they identify (with varying degrees of accuracy) those patients that should be excluded from opioid therapy, the implicit conclusion is that any patients not at risk for abuse are equally suitable candidates for this form of treatment. The authors argue, based on the review of the literature and clinical observations by those in the pain management field, that individuals who benefit physically, emotionally, and socially from COT can be identified. Furthermore, we propose that identification of these individuals goes beyond excluding those who are at risk for opioid misuse. We propose that an ideal candidate for COT would present with no evidence of aberrant opioid use, maintain good social and occupational functioning, experience manageable levels of pain, and engage in adaptive emotional regulation. Exclusion screening measures, while useful, are insufficient to differentiate among chronic pain patients. Currently, there does not exist an inclusion measure, or cohesive set of predictive factors, meant to identify patients who would likely benefit from COT. 

Operationalizing what is considered a positive outcome for any intervention for chronic pain is complicated. Pain is not only a sensory experience, but also impacts physical, emotional, and social functioning. The Initiative on Methods, Measurement and Pain Assessment in Clinical Trials (IMMPACT) has attempted to address this issue. Six core domains were identified: (a) pain; (b) physical functioning; (c) emotional functioning; (d) self-report ratings of improvement and satisfaction; (e) symptoms and adverse events; and (f) participant disposition [44]. The first five domains appear to be directly applicable to the determining success of COT. Although the extent of improvements needed in each domain is beyond the scope of this article, IMMPACT recommendations recommend improvements of at least 30% in pain and improvements, or return to normative levels, in physical and emotional functioning [45]. In general, individuals with chronic pain on opioids for long-term use would ideally have lower levels of pain as well as intact physical, emotional, social, and occupational functioning. 

### 3.3. Developing Inclusion Measures: The Biopsychosocial Approach as a Foundation

As the biopsychosocial approach is considered to be an effective model for the treatment of chronic pain [12], it represents the most comprehensive foundation from which to design screening tools [12]. The goal of such a measure would be to obtain, from multiple sources (*i.e.*, patients, laboratory tests, *etc.*), the biological, psychological, and social factors that we hypothesize might contribute to successful long-term opioid therapy. We define “successful” long-term opioid therapy to include the following: a reduction in perceived pain level and associated physical symptoms (biological); improved, or maintained, social or occupational functioning (social); and active coping and adaptive emotional regulation (psychological). 

## 4. Potential Inclusion Factors

Although one factor may not capture those who may benefit, a combination of factors may be useful. Furthermore, inclusion screening tools are not suggested to be used in place of exclusion tools, but rather as a complement. Another potential shift may need to occur with regard to the development of inclusion screening tools, namely, a shift from more “state”-like factors to those that are more “trait”-like in nature. State-like factors by definition are transitory and heavily influenced by changes in the environment, such as a shift in affect after a positive experience. Trait-like factors, although modifiable, remain relatively more stable over time, such as a tendency to work diligently in many areas of one’s life [46]. Early in the development of exclusion screening tools, an emphasis was placed on current and past substance use, current and past depressive and anxiety symptoms, and legal history, along with other state and trait factors [3,4]. States and traits were often conflated in these measures. Problems arose, as depression and anxiety are often natural consequences of pain, even in individuals without a previous history of these difficulties [47,48]. For an inclusion measure, an emphasis on trait-like characteristics may help to ameliorate the concern for fluctuating mental and emotional states. 

Below is an initial attempt to describe some of the potential factors for future research into who may benefit from COT.

### 4.1. Biological

Unlike the measures designed to detect opioid misuse, biological factors may outweigh psychosocial factors in determining who may benefit from opioids. For instance, identifying those individuals who are at low risk of opioid abuse or who develop tolerance more slowly may best be determined under the biological heading of the biopsychosocial approach. 

#### 4.1.1. Pain Condition

Previous evidence suggests that individuals who have failed non-opioid interventions and experience moderate to severe pain may be appropriate for long-term opioid use [6,29,30,49]. Individuals with arthritic, musculoskeletal, and neuropathic chronic pain, with limited psychosocial overlay, and in the absence of established opioid misuse risk factors, may constitute a minimal threshold that could be combined with remaining factors to determine who may benefit from COT [6]. 

#### 4.1.2. Genetic Factors

With the advent of more sophisticated and cost-effective techniques for genetic testing, the potential for identifying those who may benefit from COT has increased. Evidence from twin and adoption research points to a heritable vulnerability for opioid dependence [50]. For instance, Tsuang, Bar *et al.* [51] concluded that genetic factors accounted for 34% of the variance of drug use with a moderate contribution (43%) to opioid dependence. 

Evidence for the role of genetic polymorphisms is still in the early, but expanding, stages of investigation. Several potential polymorphisms have been identified that are related to opioids, including A118G, DRD2, DRD4, OPRM1, OPRD1, OPRK1, and BDNF, to name only a few [51,52]. For example, the single nucleotide polymorphism (SNP) A118G, associated with the µ opioid receptor, appears to decrease the effectiveness of morphine [52]; genes related to the cytochrome P450 (CYP450) system of the liver, CYP2D6 polymorphisms, impact metabolization of codeine [53]; and the SNP UGT2B7, associated with UDP-glucuronosyltransferases, has been found to correlate with morphine metabolization [54]. 

Along with the study of genetic polymorphisms, genome-wide linkage studies provide another opportunity to examine the genetics of opioid use. Gelernter, Panhuysen *et al.* [54] evaluated 393 related individuals with a minimum of one family member who met criteria for opioid dependence. The investigators concluded that the evidence “strongly supports a risk locus for a trait defined by symptoms related to heavy opioid use on chromosome 17” (p. 764).

### 4.2. Psychological

Potential psychological and social factors that could be examined have been explored in other populations and serve as protective factors against the impact of stress on physical, emotional, and social functioning. The authors do not propose that these factors would improve the efficacy of COT, but rather would decrease the chances of engaging in maladapative coping, such as aberrant drug behavior, avoidance of social activity, and a limiting of self-care activities (e.g., exercise). As COT has the potential to negatively impact mood and cognition [55,56], identifying and studying protective factors against such outcomes is warranted. 

#### 4.2.1. Resiliency

Resiliency has been defined as a “dynamic process encompassing positive adaptation within the context of significant adversity” [57] (p. 543), and it represents an ideal candidate factor for who may benefit from COT. The research on resiliency spans over 40 years, and the validity of this construct serving as a protective factor in the face of stress or adversity is well established [58]. For example, resiliency was found to relate to lower pain and lower negative affect over time among individuals with chronic pain [59]. Although more research is needed, resiliency within the context of chronic pain may be one factor to be considered regarding whom may benefit from COT. 

#### 4.2.2. Personality

The Big Five personality traits offer another area of potential variables that point to who may benefit from COT. The Big Five traits have a strong evidence basis with regard to the validity of the constructs and continued predictive value of individuals’ behavior over time [46]. For instance, higher levels of neuroticism have been associated with higher levels of pain and lower levels of functioning [60]. Furthermore, higher scores on extraversion [61], openness [62], and conscientiousness [63] have been found to be related to more active coping among individuals with chronic pain. With the exception of neuroticism, which could be seen as an exclusion factor, extraversion, openness, and conscientiousness represent potential inclusion factors. 

### 4.3. Social

#### 4.3.1. Social Support

The concept of social support is a widely recognized mediating factor with regard to stress [64] and represents another potential inclusive factor regarding who may benefit from COT. An intact social support system, as well as an ability to make use of that support system, decreases the negative impact of stress, both physically and emotionally [65]. In a study with 78 rheumatoid arthritis patients who were followed for five years, the authors concluded that low levels of social support impacted disability and pain levels over the course of a five-year period [66]. However, social support should be considered one factor, and it is quite possible that individuals with limited social support could benefit from COT. 

#### 4.3.2. Employment

The nature and context of employment may also play a role in the determination of who benefits from COT. Employment serves as a stabilizing force with regard to identity and socioeconomic stress [67]. Although much has been written about the negative impact of the loss of one’s job or the potentially damaging effects of the workers’ compensation or disability systems [67], a relevant construct in this area relates to “secondary loss” [68]. Specifically, this concept refers to the types of losses that occur when employment is lost or significantly diminished. Furthermore, research provides evidence for the health benefits of employment [69], and this broad area provides several potential inclusion factors concerning who may benefit from COT, including job satisfaction and stability of employment. As with social support, this area requires further research and individuals who are unemployed after a work-related injury may benefit from COT. 

## 5. Directions for Future Research and Conclusions

This review article has attempted to present an alternative perspective on the question of opioid misuse risk with the goal of spurring additional research into who may benefit from COT. We propose that focusing on opioid misuse risk may inadvertently lead to the assumption that individuals who are not at high risk of misusing opioids would therefore benefit from COT. Identifying those at risk and those who benefit can be seen as complementary areas of inquiry. Additional research is needed to: (a) operationalize what constitutes a positive outcome for individuals on COT; (b) identify and evaluate those who have been maintained on low doses of opioids with good pain control and functioning; and (c) prospectively evaluate potential biological, psychological, and social factors that may predict who benefits from COT. Although qualitative research is often disparaged by those engaged in quantitative research efforts, the importance of qualitative research cannot be understated in a nascent area of study with regard to generating a hypothesis for future quantitative evaluation. We argue that this line of investigation would benefit from both qualitative and quantitative research endeavors in the future.
